# Exosomal tau with seeding activity is released from Alzheimer’s disease synapses, and seeding potential is associated with amyloid beta

**DOI:** 10.1038/s41374-021-00644-z

**Published:** 2021-08-30

**Authors:** Emily Miyoshi, Tina Bilousova, Mikhail Melnik, Danyl Fakhrutdinov, Wayne W. Poon, Harry V. Vinters, Carol A. Miller, Maria Corrada, Claudia Kawas, Ryan Bohannan, Chad Caraway, Chris Elias, Katherine N. Maina, Jesus J. Campagna, Varghese John, Karen Hoppens Gylys

**Affiliations:** 1 UCLA School of Nursing, UCLA School of Medicine, Los Angeles, CA USA; 2 Mary S. Easton Center for Alzheimer’s Research at UCLA, UCLA School of Medicine, Los Angeles, CA USA; 3 Department of Neurology, UCLA School of Medicine, Los Angeles, CA USA; 4 Neuroscience Interdepartmental Program, UCLA School of Medicine, Los Angeles, CA USA; 5 Institute for Memory Impairments and Neurological Disorders, UC Irvine, Irvine, CA USA; 6 Department of Pathology and Laboratory Medicine, UCLA School of Medicine, Los Angeles, CA USA; 7 Department of Pathology, Keck USC School of Medicine, Los Angeles, CA USA; 8 Department of Neurology, Program in Neuroscience, Keck USC School of Medicine, Los Angeles, CA USA; 9 Department of Neurology, UC Irvine, Irvine, CA USA; 10 Department of Neurobiology and Behavior, UC Irvine, Irvine, CA USA

**Keywords:** Alzheimer's disease, Alzheimer's disease

## Abstract

Synaptic transfer of tau has long been hypothesized from the human pathology pattern and has been demonstrated in vitro and in vivo, but the precise mechanisms remain unclear. Extracellular vesicles such as exosomes have been suggested as a mechanism, but not all tau is exosomal. The present experiments use a novel flow cytometry assay to quantify depolarization of synaptosomes by KCl after loading with FM2–10, which induces a fluorescence reduction associated with synaptic vesicle release; the degree of reduction in cryopreserved human samples equaled that seen in fresh mouse synaptosomes. Depolarization induced the release of vesicles in the size range of exosomes, along with tetraspanin markers of extracellular vesicles. A number of tau peptides were released, including tau oligomers; released tau was primarily unphosphorylated and C-terminal truncated, with Aβ release just above background. When exosomes were immunopurified from release supernatants, a prominent tau band showed a dark smeared appearance of SDS-stable oligomers along with the exosomal marker syntenin-1, and these exosomes induced aggregation in the HEK tau biosensor assay. However, the flow-through did not seed aggregation. Size exclusion chromatography of purified released exosomes shows faint signals from tau in the same fractions that show a CD63 band, an exosomal size signal, and seeding activity. Crude synaptosomes from control, tauopathy, and AD cases demonstrated lower seeding in tauopathy compared to AD that is correlated with the measured Aβ42 level. These results show that AD synapses release exosomal tau that is C-terminal-truncated, oligomeric, and with seeding activity that is enhanced by Aβ. Taken together with previous findings, these results are consistent with a direct prion-like heterotypic seeding of tau by Aβ within synaptic terminals, with subsequent loading of aggregated tau onto exosomes that are released and competent for tau seeding activity.

## Abstract (web summary)

A novel assay quantifies depolarization of synaptosomes from Alzheimer’s cortex, and the authors show that tau released from these samples is unphosphorylated and C-terminal-truncated. Most tau is exosomal, and free-floating tau did not seed aggregation. Seeding activity was lower in tauopathy than AD, and strongly related to amyloid level.

## Introduction

Of the two hallmark lesions of Alzheimer’s disease (AD), the aggregated tau protein in neurofibrillary tangles correlates best with cognitive dysfunction. In AD, NFT pathology spreads in a stereotypic pattern, beginning in entorhinal cortex, moving to the hippocampus, and eventually to neocortex^1^. For this reason, trans-synaptic spread of tau pathology between regions has long been hypothesized and more recently been shown convincingly in vitro^2–4^ and in vivo^5^. The importance of the synapse in transfer is highlighted by the result that synaptic contacts are required for exosome-mediated transmission of tau in vitro^6^, and increased activity stimulates release and enhances tau pathology in vivo^7^. Along this line, work from our lab has shown that tau protein is abundant in control and AD synapses, and is released by in vitro depolarization of AD synaptosomes^8^.

Exosomes are extracellular vesicles (EVs) of endocytic origin, usually defined by a size range ~30–120 nm, and by content of endosome-associated proteins^9^. Recent evidence suggests exosomes as a mechanism for prion-like spread of misfolded proteins in neurodegeneration^10,11^, and exosome-associated spread of tau pathology has been shown in the rTg4510 mouse and several other model systems^6,12,13^. However, released tau is found to be free-floating as well as localized to plasma-membrane-derived EVs, microvesicles, or ectosomes^14^, and the degree of exosomal localization varies with model system^13,15^. Similarly, results vary with respect to the degree of phosphorylation and truncation of released tau^6,12,16,17^. The size of the tau aggregate also affects propagation, with trimers being a minimal unit for seeding aggregation^18^; in another study, soluble high molecular weight (HMW) p-tau peptides were rare, but were the key species for propagation^19,20^. Like amyloid β (Aβ), tau protein forms oligomeric intermediates that mediate downstream toxicity; we and others have shown tau oligomers localized to synapses^21–23^. Aβ is also found in exosomes, and has been shown to propagate by exosomes^24^. Blockade of exosomal pathways has been shown to reduce plaque pathology^25^, and suggested as a possible therapeutic approach for reduction of tau propagation^26^. Because tau, p-tau, and Aβ are elevated in brain-derived exosomes from plasma, exosomes have also been proposed as blood-based biomarkers for AD^27^.

Aβ immunotherapy has generally not shown efficacy in humans, but both active and passive tau immunotherapy has shown encouraging results in at least 13 studies in animal models^16,28,29^; tau immunotherapy has been shown to reduce amyloid as well as tau pathology^30^. However, a primary challenge for tau-based immunotherapy approaches is the lack of understanding of the precise form of the tau peptide(s) that is neurotoxic^28^. Based on our previous work using cryopreserved postmortem AD synaptosomes and the lack of consensus in the literature, we have developed an assay to quantify depolarization of in vitro synaptosomes, and demonstrated depolarization-induced release of tau and exosome-like EVs from AD synaptosomes. Released tau peptides from AD synapses are largely C-terminal-truncated, unphosphorylated, and oligomeric. Exosomal tau and tau oligomers are increased in AD compared to control exosomes, and exosomes released from AD synapses demonstrate elevated seeding activity that is enhanced by Aβ in a FRET-based biosensor assay.

## Materials and methods

### Human brain specimens

The primary goal of the present experiments was to characterize tau and exosomes released from AD synaptosomes; due to the requirement for large sample volume, only a few control brains were used for key experiments. Pilot experiments showed a higher degree of depolarization for samples with relatively short postmortem interval (PMI) (<6 h); therefore samples were chosen based on PMI and relatively similar levels of AD pathology. Frontal and parietal cortex (Brodmann areas A7, A9, A39, A40) samples were obtained at autopsy from AD research centers at UCLA, UCI, and USC (Table 1). Immediately on receipt, samples (~0.3–5 g) were minced in a 0.32 M sucrose solution with protease inhibitors for cryopreservation of synaptic structure and membranes^31^ (2 mM EDTA, 2 mM EGTA, 0.2 mM PMSF, 1 mM Na pyrophosphate, 5 mM NaF, 10 mM Tris), then stored at −80 °C until homogenization. The P-2 (crude synaptosome; synaptosome-enriched fraction) was prepared as previously described^32^; briefly, tissue was homogenized in ice cold buffer (0.32 M sucrose, 10 mM TRIS pH 7.5, plus protease inhibitors: pepstatin (4 μg/ml), aprotinin (5 μg/ml), trypsin inhibitor (20 μg/ml), EDTA (2 mM), EGTA (2 mM), PMSF (0.2 mM), Leu-peptin (4 μg/ml)). The homogenate was first centrifuged at 1000 *g* for 10 min; the resulting supernatant was centrifuged at 10,000 *g* for 20 min to obtain the crude synaptosomal pellet. Aliquots of P-2 are routinely cryopreserved in 0.32 M sucrose and banked at −80 °C until the day of the experiment^31^, at which time they were defrosted at 37 °C.

**Table 1:** Case information.

Case number	Sex	Age	PMI, h	Braak stage	Aβ plaque A39/40	Diagnoses	Figures using case
Alzheimer’s disease							
11-09	M	77	5.5	VI	Moderate	AD/atherosclerosis/CAA	Figs. 1d and 2a, b
11-17	F	69	3.6	VI	Frequent	AD/CAA	Fig. 2d
12-12	F	82	7	VI	Frequent	AD	Fig. 5 (AD8)
12-13	F	95	6.2	VI	Frequent	AD/CAA/vascular dementia	Fig. 4
13-14	F	63	6.5	VI	Frequent	AD/hippocampal sclerosis/CAA	Fig. 4
16-12	F	≥90	5.16	VI	Moderate	AD/CAA	Fig. 2a, b
16-15	F	55	5.57	VI	Frequent	AD/arteriolar sclerosis/CAA/hippocampal sclerosis	Fig. 3d
17-18	M	81	6.58	VI	Moderate	AD/atherosclerosis/CAA/vascular dementia	Fig. 4
2-12	F	64	5.3	V	Frequent	AD/CAA	Fig. 2a, b
21-11	M	96	5.4	VI	Frequent	AD/atherosclerotic leukoencephalopathy/arterial sclerosis/CAA/hippocampal sclerosis	Fig. 4
21-12	M	90	3.5	V	Moderate	AD/atherosclerosis/hippocampal sclerosis	Fig. 4
21-17	M	≥90	5.32	V	Moderate	AD/atherosclerosis/hemorrhages	Fig. 2d
22-16	M	90	5.42	VI	Moderate	AD/atherosclerosis/hemorrhage	Fig. 4
22-17	M	90	6.08	V	Sparse	AD/atherosclerosis/CAA/Lewy bodies (Amygdala)	Fig. 4
22-18	F	89	5.25	VI	Moderate	AD	Fig. 4
23-11	M	71	6.2	IV	Frequent	AD	Fig. 5 (AD7)
24-12	F	≥90	7.41	V	Sparse	AD/arterial sclerosis/atherosclerosis/hippocampal sclerosis/solitary infarct	Fig. 2a, b
24-17	F	≥90	4.43	VI	Frequent	AD/CAA	Fig. 2d
25-10	F	57	3.55	VI	Frequent	AD	Figs. 1d, 2d, and 3c (AD5)
28-11	M	81	4	VI	Moderate	AD/CAA/multiple infarctions/trauma	Figs. 1d and 2d
29-15	M	83	4.73	V	Moderate	AD/CAA	Fig. 2d, e
3-12	M	81	5	IV	Sparse	AD/atherosclerosis	Fig. 2b
3-13	F	≥90	4.66	VI	Frequent	AD/diffuse LB/hippocampal sclerosis	Figs. 1d, 2a, b, d, and 3a, c (AD1 for Fig. 3)
3-16	F	72	5.42	IV	Frequent	AD	Fig. 2d
30-10	M	46	5.3	VI	Moderate	AD/CAA/vascular dementia	Figs. 1d and 2d
31-11	F	≥90	3.55	V	Frequent	AD/hippocampal sclerosis	Fig. 2a, b, d
33-09	M	93	6.36	VI	Frequent	AD/arteriolar sclerosis/atherosclerosis/CAA	Fig. 4
33-14	F	73	5.75	VI	Frequent	AD	Fig. 2a and b
34-12	F	≥90	5	VI	Sparse	AD/CAA/hippocampal sclerosis	Fig. 2d
34-13	M	46	4.6	VI	Frequent	AD/CAA/vascular dementia	Fig. 2a and b
37-10	F	88	5.3	V	Frequent	AD/subcortical arteriosclerotic leukoencephalopathy	Figs. 2d and 4
37-12	M	≥90	5.41	VI	Moderate	AD/arteriosclerosis/atherosclerosis/micro-hemorrhages	Figs. 1a–d and 2d, e
37-15	F	87	6.03	VI	Frequent	AD/atherosclerosis/meningioma	Fig. 4
38-11	M	≥90	5.5	V	Moderate	AD	Figs. 1d and 2a, b, d
42-16	F	74	4.53	V	Frequent	AD/CAA/Diffuse LB	Fig. 4
47-16	F	≥90	6.92	VI	Moderate	AD	Figs. 1d and 2d
48-17	F	92	5.03	VI	Moderate	AD/atherosclerosis	Figs. 3c, e and 4
5-13	F	≥90	4.92	V	Frequent	AD/atherosclerosis/CAA/vascular dementia	Figs. 1g, 2a, b, d, and 3a, c (AD2 for Fig.3)
6-14	F	64	6.15	VI	Moderate	AD	Fig. 2d
8-13	M	≥90	4.85	V	Sparse	AD/atherosclerosis/hippocampal sclerosis	Fig. 2d, e
805	F	≥90	8.5	V	Frequent	AD/arteriosclerosis/atherosclerosis/CAA	Fig. 1d
811	M	59	5.5+	VI	Frequent	AD/CAA/hydrocephalus	Fig. 2a, b, d
813	M	79	5.75	V	Frequent	AD/CAA	Fig. 2a, b
869	F	75	5	VI	Moderate	AD	Fig. 2a, b
871	F	88	9	V	Sparse	AD/CAA/cerebral contusion/hippocampal sclerosis	Fig. 2a, b
9-18	F	89	4.87	V	Frequent	AD/ischemic leukoencephalopathy/atherosclerosis/vascular dementia	Fig. 4
900	M	87	5.5	V–VI	Frequent	AD/CAA	Figs. 1d, 1e, f, and 2d
909	M	86	5.5+	V	Frequent	AD/CAA/ependymitis	Fig. 2d
U1	F	96	5	VI	Frequent	AD/CAA/cerebrovascular disease	Fig. 2d
U2	M	82	5	V–VI	Moderate	AD/CAA/cerebrovascular disease	Figs. 1d and 2a, b
7-11	F	≥90	4.25	III	Moderate	CIND/atherosclerosis	Fig. 5 (AD6)
Tauopathy							
19-12	M	≥90	5.91	III	None	Hippocampal sclerosis	Fig. 5 (T3)
20-12	F	≥90	7.5	IV	Sparse	Hippocampal sclerosis	Fig. 5 (T4)
23-12	F	≥90	6	III	None	CAA/solitary infarct/hippocampal sclerosis	Fig. 5 (T2)
31-12	M	≥90	6	IV	None	Hippocampal sclerosis	Fig. 5 (T5)
U3	M	75	8	IV	None	NFT-predominant AD/arteriosclerosis/atherosclerosis	Fig. 5 (T1)
Controls							
1-13	M	≥90	6	I	None	Atherosclerosis/solitary infarct	Fig. 5 (N1)
830	F	89	4.25	II	None	AD/vascular dementia/atrophy	Fig. 3a (Con)
907	M	84	5	N/A	None	Normal	Fig. 5 (N2)

Plaques number in the area (based on Bielschowsky stain and IHC): none = 0; sparse = 1–5; moderate = 6–20; frequent = more than 20.

*CIND* cognitive impairment, no dementia, *CAA* cerebral amyloid angiopathy.

### Mouse brain specimens

WT mice expressing human apoE (E3 and E4) from a previous study were euthanized, and cortices (~0.1–0.15 g) were processed into P-2 immediately. In order to examine differences between fresh and frozen samples, mouse P-2 samples were not cryopreserved and were immediately used for our depolarization assay.

### Depolarization assay

For preparation of buffers used in our depolarization assay, ASTM type 1 water (LabChem) was used. After defrosting, human P-2 samples were centrifuged at 10,000 *g* for 10 min at 4 °C to remove sucrose; typical experiments used two 0.3 ml aliquots (~25.8 mg tissue). Samples were then resuspended in Normal Krebs buffer (160 mM NaCl, 5.5 mM KCl, 10 mM HEPES, 10 mM glucose, 10 mM pyruvate, 1.2 mM MgCl_2_, 1.5 mM CaCl_2_) and incubated at 37 °C for 5 min. Samples were divided in order to measure depolarization with FM2–10; simultaneously, release fractions were collected for biochemical analysis and exosome purification. Based on a previous protocol for FM2–10 ^33^, P-2 aliquots (100 μl) were incubated with FM2–10 (25 μM) for a minute before a 2 min 30 mM KCl stimulation at 37 °C. Excess dye was then washed out with 1 mg/ml BSA, and the samples were centrifuged at 10,000 *g* for 10 min at room temperature. A second wash was performed with Normal Krebs before resuspending and dividing each sample between two flow cytometry tubes. Fifty mM final KCl was added to one tube, and both tubes were read on BD FACSCalibur (Becton-Dickinson, San Jose, CA) at 5, 10, and 20 min timepoints. FM2–10 and calcein AM fluorescence was plotted against forward scatter, which is proportional to size. An analysis gate was drawn on forward scatter (FSC), based on size standards (0.75–1.5 μm), to ensure that fluorescence was quantified only in particles within the size range of synaptosomes^34,35^. Flow cytometry data were analyzed using FCS Express version 5 software (DeNovo Software California, USA).

The remaining P-2 samples for biochemistry/exosome isolation were incubated at 37 °C for 3 min and centrifuged. Samples were resuspended in Normal Krebs and divided between two tubes. Equal volumes of Normal Krebs and 50 mM final KCl were added to corresponding tubes and incubated for 5 min at 37 °C before immediate addition of protease and phosphatase inhibitors (Fisher, Waltham, MA) followed by centrifugation at 10,000 × *g* at 4 °C. Supernatants were collected and either immediately use for fractionation/concentration with Vivaspin 500 centrifugal concentrators (Sartorius, Gottingen, Germany) and/or exosome isolation or stored at −80 °C. Total protein concentration was determined with the Pierce BCA assay.

### Western and dot blotting

Collected supernatants were separated by gel electrophoresis on 10–20% Tris-Glycine gradient gels with 4x Tris-Glycine sample buffer. After transferring to Immobilon-P membrane, membranes were blocked with 5% BSA for 1 h, and primary antibodies were incubated overnight at 4 °C. Antibodies used are listed in Table 2. For dot blotting, samples were applied to a nitrocellulose membrane using the Bio-Dot microfiltration apparatus. The membrane was blocked with 5% milk for 1 h, and the primary antibody incubated overnight at 4 °C. After secondary antibody incubation, immunolabeled proteins were visualized and quantified by SuperSignal West Femto maximum sensitivity substrate (Thermo Scientific, Rockford, IL) on a UVP BioSpectrum 600 imaging system using VisionWorks Version 6.6A software (Upland, CA). Human recombinant tau441 protein preformed fibrils (PFF) used in control experiments were purchased from StressMarq Biosciences (SPR-475B, Victoria, Canada); for each experiment the aliquot was divided in two equal parts and 1/2 was sonicated for 10 min in water bath sonicator therefore the added tau contained a mixture of different size tau fibrils and oligomers.

**Table 2:** Reagents.

Antibody name	Antigen/epitope	Supplier	Host	Reactivity
FM2–10 (dye)	Associates with cellular membranes	Invitrogen (Waltham, MA)	N/A	N/A
Calcein AM (dye)	Intact cells/synaptosomes (viability dye)	Biolegend (San Diego, CA)	N/A	N/A
Exosme-anti-CD63 for western (TS63)	CD63	Invitrogen (Waltham, MA)	Mouse	Human
Exosome-anti-CD81 for Western (M38)	CD81	Invitrogen (Waltham, MA)	Mouse	Human
Exosome-anti-CD9 for western (TS9)	CD9	Invitrogen (Waltham, MA)	Mouse	Human
Anti-HSP70 antibody	HSP70	SBI System Biosciences (Palo Alto, CA)	Rabbit	Human
Tau monoclonal antibody (HT7)	Tau (including PHF and non-PHF tau)	Invitrogen (Waltham, MA)	Mouse	Human, Bovine
Phospho-Tau (pS422) polyclonal antibody	Tau (phospho-serine 422 specific)	Invitrogen (Waltham, MA)	Rabbit	Human
Anti-Tau antibody, clonal Tau12	Tau (N-terminal-specific)	Biolegend (San Diego, CA)	Mouse	Human
Tau antibody (T46)	Tau (C-terminal-specific)	Invitrogen (Waltham, MA)	Mouse	Human, Non-Human Primate, Mouse, Rat,
Anti-Tau(22), oligomeric antibody	Oligomeric Tau	Millipore (Burlington, MA)	Rabbit	Human
Recombinant anti-syntenin antibody	Syntenin-1	Abcam (Cambridge, UK)	Rabbit	Human

### Transmission electron microscopy (TEM)

Collected supernatants from the depolarization assay were concentrated using Vivaspin 500 centrifugal concentrators (Sartorius, 100,000 MWCO). The concentrate was fixed on a copper mesh in glutaraldehyde/paraformaldehyde solution followed by staining with 2% uranyl acetate solution and imaged on JEOL 100CX electron microscope at ×29,000 magnification.

### Exosome isolation: immunoprecipitation

Antibodies for three general exosomal surface markers: tetraspanins CD63 (TS63), CD9 (TS9), and CD81 (M38) from Invitrogen were first coupled to superparamagnetic beads (Dynabeads MP-270 Epoxy; ThemoFisher) using a dynabeads antibody coupling kit (ThermoFisher), according to the manufacturer’s instructions. To ensure maximal exosomal yield during immunoprecipitation (IP), anti-CD63, anti-CD9, and anti-CD81, beads were added simultaneously to the samples in final concentration 0.1 mg/ml for each type of beads (pan-specific isolation) followed by overnight rotation at 4 °C. After four washes with PBS, exosomes were lysed by 10 min boiling in 50 µl of 1x Novex Tris-Glycine SDS Sample buffer with DTT (200 µM). Beads were separated by magnet and IP samples were run in 10–20% SDS PAGE (Invitrogen) followed by immunoblotting analysis with syntenin-1 (H-48, Santa Cruz Biotech) and tau (HT7, Invitrogen) antibodies. Standard human plasma exosomes were purchased (Galen Laboratory Supplies, Middletown, CT), and loaded as a positive control.

### Exosome isolation: ultracentrifugation

The supernatant was spun at 2000 × *g* for 10 min, then the supernatant was collected and spun at 10,000 × *g* for 30 min at 4 °C. Supernatant from the last centrifugation was applied to a triple sucrose cushion gradient for ultracentrifugation (80,000 × *g* for 3 h at 4 °C). Fractions 1–3 were collected as described^36^, and diluted for a final spin (100,000 × *g* for 1 h at 4 °C) to pellet vesicles, then suspended in 25 mM trehalose/PBS to prevent aggregation and cryodamage, then slow frozen at −80 °C. EV size was determined using tunable resisting pulse sensing analysis using a qNano Gold instrument using nanopore NP100 (Izon Science, Medford, MA).

### Exosome isolation: size exclusion chromatography (SEC)

Supernatants collected after synaptosome depolarization (150 or 300 µl of supernatants from P-2 weight equal 0.04 g or 0.12 g, respectively) were loaded to prewashed qEVsingle/35 nm SEC columns (Izon Science, Medford, MA) and fraction were collected according to manufacturer’s instructions. Briefly, after void volume (1 ml) was discard, five fractions of 200 µl each (F5, F6, F7, F8, and F9) were collected. Halt’s protease and phosphatase inhibitor cocktail (ThermoFisher, Waltham, MA) were added and the fractions were aliquoted and kept at −80 °C. For control experiments one aliquot of tau PFF (StressMarq Biosciences, Victoria, Canada), contained 10 µg of tau PFF in 10 µl PBS, was sonicated for 10 min and then mixed with an aliquot of tau PFF (which was not sonicated) and 20 µg of standard human plasma exosomes (Galen Laboratory Supplies, Middletown, CT), volume of mixture was adjusted to 150 µl and loaded to qEVsingle/35 nm SEC columns and fractions were collected as described above. In parallel, the same amount of tau PFF (20 µg) was processed in an exact same way but was not mixed with exosomes before applying to a qEV column.

### Tau biosensor cell assay

We used the HEK293T Tau RD P301S FRET biosensor (tau biosensor) assay according to the previously described protocol^37^. Briefly, samples were sonicated for 10 min and transduced to tau biosensors using Lipofectamine 2000 (ThermoFisher Scientific, Walthman, MA). Cells were harvested at the 60 h timepoint and fixed using 2% solution of paraformaldehyde. An Attune NxT Flow Cytometer (Invitrogen) equipped with an autosampler and FRET-compatible laser lines and filter sets was used for FRET signal detection. The FRET (CFP/YFP) signal was excited by a 405 nm laser for CFP excitation and detected in the YFP image detection channel. Flow cytometry data were analyzed using FCS Express version 5 software (DeNovo Software California, USA). Integrated FRET density was calculated as a product of percentage FRET positive cells and median of fluorescent intensity of the FRET positive cells as previously established^37^.

### Statistical analysis

Data are presented as mean ± SEM. All comparisons between control and depolarized samples used a Student’s *t* test for paired samples unless otherwise noted. One-way analysis of variance with Tukey HSD post hoc test was used for experiments with three or more comparison groups.

## Results

### The styryl dye FM2–10 quantifies synaptosome depolarization in vitro

**Fig. 1:**
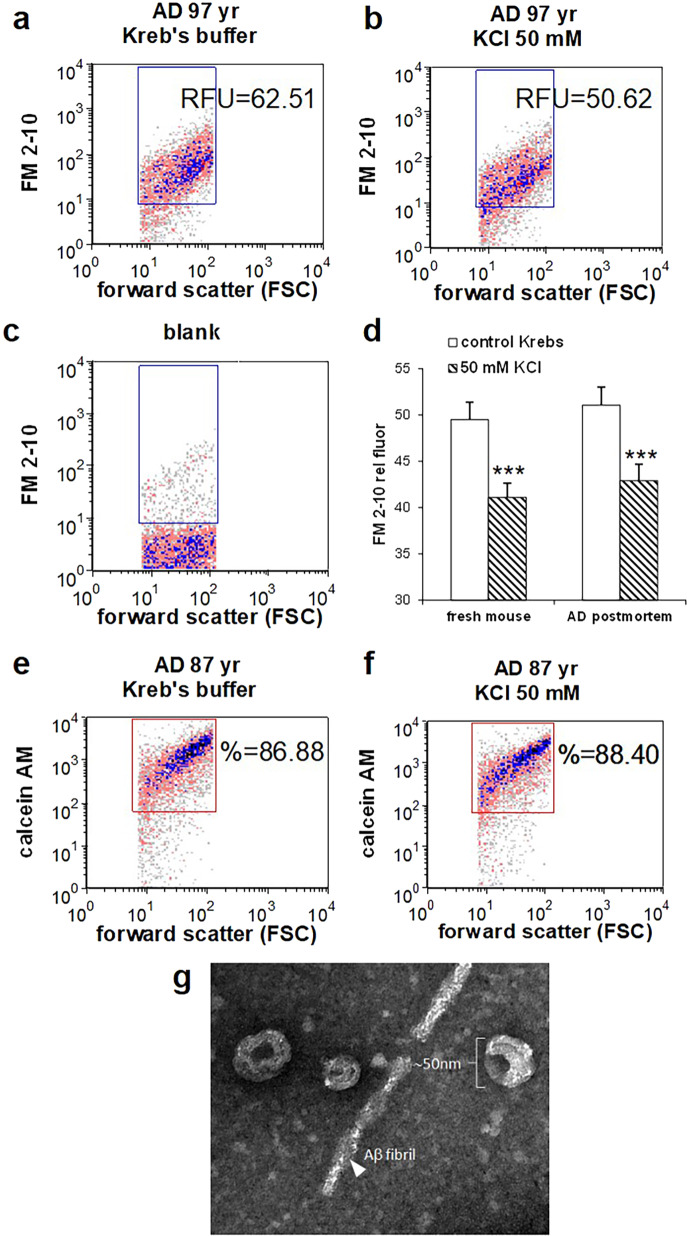
Flow cytometry assay for in vitro quantification of depolarization. AD cortical synaptosomes were incubated with FM2–10 (25μM) prior to incubation with KCl (30 mM) and flow cytometry analysis. Representative density plots illustrate FM2–10 labeling in baseline (**a**) and depolarized samples (**b**); reduction in fluorescence corresponds to exocytic activity. Forward scatter (FSC) is proportional to particle size; rectangular analysis gate is drawn on size standard to include particles from ~0.5 to 1.5 μm, data collected from 5000 events. **c** Background labeling in unstained blank. **d** Aggregate data from human AD cortex (A7 or A9; *n* = 13) and fresh mouse cortex (*n* = 12; *p* < 0.0001). **e** Representative density plots illustrate viability dye calcein AM, showing integrity of baseline (**e**) and depolarized (**f**) P-2 samples. **g** AD release supernatant was concentrated as described in “Materials and methods” for transmission electron microscopy.

Synaptosomes are resealed nerved terminals prepared from fresh brain tissue that contain mitochondria and maintain membrane potential, along with a full complement of receptor and transporter functions^38^. Functional endpoints such as LTP and glucose transport can also be measured in postmortem human synaptosomes, if prepared from tissue that is cryopreserved by mincing and slow freezing in isotonic sucrose^39–43^. In order to verify and quantify depolarization in postmortem AD synaptosomes, we used FM2–10, a styryl dye with a lipophilic tail that becomes fluorescent on insertion into membranes^44,45^. Based on a previous exocytosis assay^33^, we developed a flow cytometry assay to measure synaptosome depolarization in vitro. P-2 samples (crude synaptosomes, synaptosome-enriched fraction) prepared from cryopreserved AD cortex were first loaded with FM2–10 dye, then depolarization was detected as a reduction in FM2–10 fluorescence reflecting vesicle release after depolarization. Dot plots present FM2–10 fluorescence vs. FSC, which is the light scattering parameter proportional to size; the blue rectangular gate is drawn based on size standards to include only particles in the size range of synaptosomes^34^. The assay is illustrated by representative cortical AD samples showing baseline fluorescence in control Kreb’s buffer (62.51 RFU; Fig. 1a), which was reduced to 50.62 by the addition of 30 mM KCl to the buffer for 5–20 min (Fig. 1b). Figure 1c illustrates typical background labeling. The mean maximum fluorescence was reduced from 51.1 to 42.9 RFU (~16%; *p* < 0.0001; Fig. 1d). Because the experimental goal was clarification of synaptically released exosomes in AD, late stage AD cases were used for initial experiments (Braak stage V–VI; Table 1) with a mean PMI 5.3 h (±0.4). To serve as non-AD controls, and to determine if the cryopreservation step for human samples affects subsequent depolarization, we next measured depolarization of mouse synaptosomes prepared immediately after sacrifice. FM2–10 fluorescence levels for baseline and depolarized mouse cortical synaptosomes were very similar to results with human samples, with a mean maximum baseline of 49.52 RFU that was reduced to 41.12 by depolarization (*p* < 0.0001; Fig. 1d). This result indicates that sucrose cryopreservation of minced tissue at the time of autopsy preserves membrane potential and exocytotic function to a level comparable to that measured in fresh synaptosomes.

To ensure that in vitro incubation with 30 mM KCl does not compromise synaptosomal membrane integrity and function, we next tested integrity of depolarized synaptosomes by labeling synaptosomes with the live cell marker calcein AM. Calcein AM is a lipophilic dye that enters cells readily and is converted to a polar fluorescent product by esterases; the dye is retained by synaptosomes with an intact membrane^46^. As shown in representative samples (Fig. 1e, f), calcein AM brightly labels ~90% of size-gated synaptosomes, and the positive fraction is not reduced by depolarization for 20 min (37 °C; 86.53 ± 0.58 % pos vs. 87.15 ± 1.3% pos). These results demonstrate the integrity of in vitro synaptosomes in our flow cytometry assay, and precisely quantify the degree of synaptosomal depolarization.

### Depolarization induces release of EVs and tau from cortical synaptosomes

**Fig. 2:**
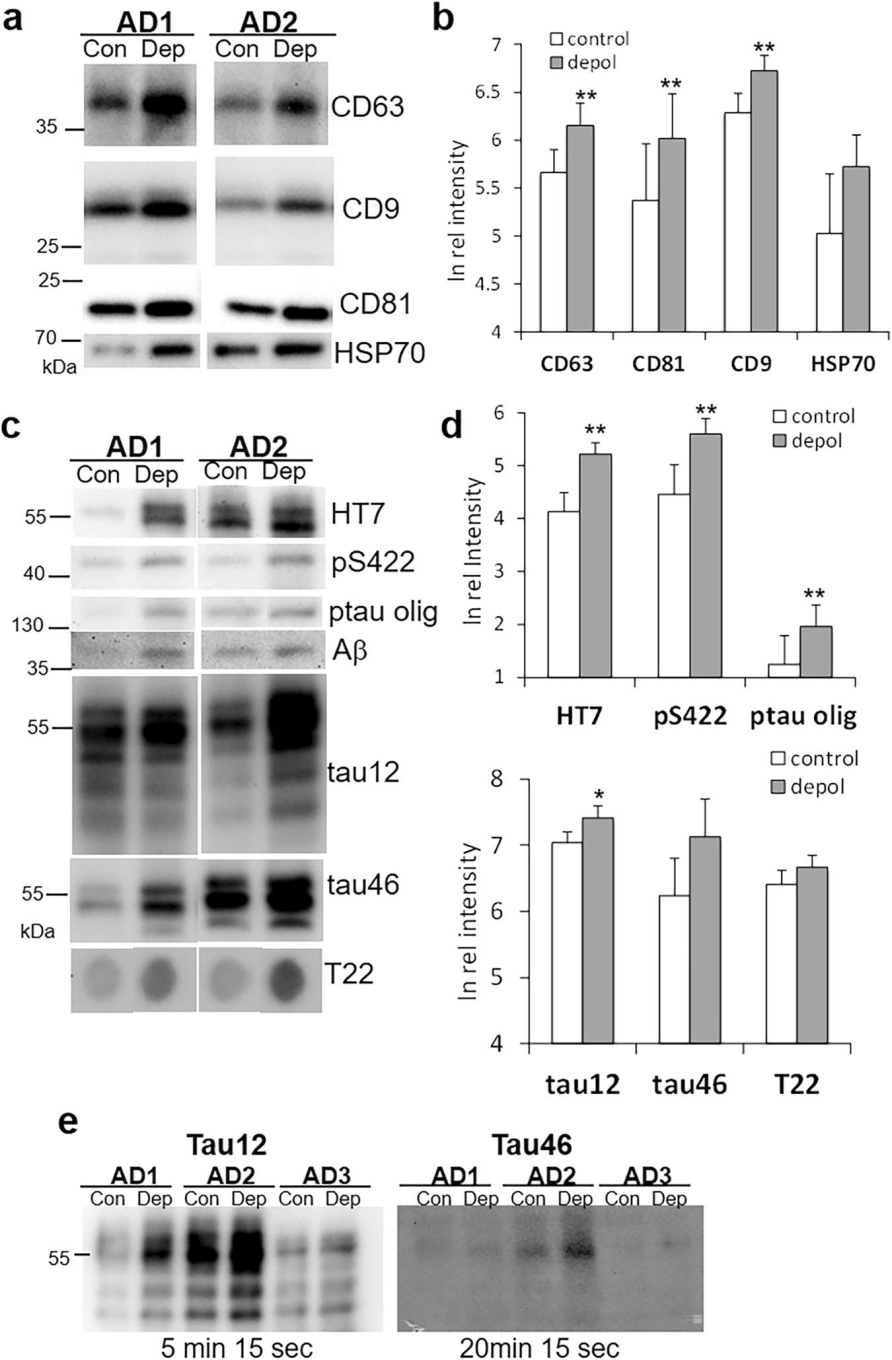
Western SDS PAGE analysis of synaptosome release supernatants. **a** Representative immunoblots demonstrate labeling for markers of extracellular vesicles: levels of the tetraspanins CD63, CD9, CD81, and heat shock protein 70 (HSP70) are compared in control (con) and depolarized (dep) samples. **b** Aggregate data for (**a**); *n* = 7, *p* < 0.01. **c** Representative immunoblots for tau peptides (see Table 1 for antibodies); the p-tau oligomer was immunolabeled with PS422. For T22 image is a dot blot. **d** Aggregate data for (**c**); *n* = 6-13, *p < 0.05, **p < 0.01. **e** Immunoblots with tau12 (detects C-terminal truncated; intact N-terminus), and tau46 (detects N-terminal truncated; intact C-terminus) that include exposure times below blot, showing faint tau46 despite a fourfold longer exposure time. Blots shown are representative of five separate experiments with 3–7 cases/blot for each of the two antibodies.

Much evidence supports EV-mediated spread of tau pathology, and the tetraspanin protein family in particular is not only highly enriched in EVs, but shown to play a role in biogenesis, assembly, and recruitment of cargos to EVs^47^. Therefore, we examined synaptosome release supernatants from AD cortex for exosomes and for tetraspanin markers of EVs. Electron microscopy confirmed that EVs released from depolarized AD cortical synaptosomes demonstrate the morphology and relative size of exosomes (30–120 nm^9^; Fig. 1g). As shown in Fig. 2a, b, the tetraspanin markers for EVs, CD63, CD9, and CD81 are all increased following depolarization (*p* < 0.01; Student’s paired *t* test). The chaperone HSP70, sometimes used as an exosome marker, showed the same trend but was not significantly elevated by depolarization.

Tau is frequent exosome cargo, and a number of papers demonstrate the release of exosomal and free-floating tau in in vitro and in vivo tauopathy models^6,12,13^; therefore we next examined the nature of tau peptides released from AD cortical synaptosomes. Consistent with previous results, the mid-region tau antibody HT7 labels most tau peptides and showed a relatively robust release of tau from synaptosomes (Fig. 2c, d; *p* < 0.01). The p-tau antibody pS422 shows that some released tau is phosphorylated, and also reveals a low level of phosphorylated tau oligomers the approximate size of a tau trimer (p-tau olig; Fig. 2c, d). However, multiple attempts to detect other p-tau epitopes (acetylated tau, caspase-cleaved tau, PHF1, p396, AT100) were not successful.

### Most tau released from AD synapses is C-terminal-truncated

The end-specific antibody tau 12 labels C-terminal-truncated tau (i.e., intact N-terminus), and tau46 labels N-terminal-truncated tau (i.e., intact C-terminus); release of both peptides was increased by depolarization (Fig. 2c, d; *p* < 0.01). Dot blots using the tau oligomer antibody T22 shows that tau oligomers are released from synaptosomes (Fig. 2c), confirming oligomeric bands observed in some samples with HT7 and tau46 (not shown); however, the depolarization-induced release trend is not significant. The aggregate data for EV markers (Fig. 2b, c) and for tau peptides (Fig. 2c, d) indicate that significant release of exosomes and tau occurs in resting synaptosomes, i.e., the increase induced by depolarization is fairly modest.

Aβ has also been reported as an exosome cargo^24,48^, therefore multiple experiments measured Aβ in AD release supernatants. Several Western blots showed faint Aβ bands on long exposure (Fig. 2c); in multiple experiments, the band was observed ~38 kDa, which is approximately the size of an apoE/Aβ heterodimer, or possibly an Aβ oligomer the size of 8-mer, both previously observed^49^. However, Aβ release was not enhanced by depolarization, and in AlphaLISA experiments, the signal was just above background, confirming the very low level of released Aβ (not shown). As noted above, both N-terminal and C-terminal tau were released from AD synaptosomes; however, comparison of blots together with exposure times (Fig. 2e) shows a very faint full-length signal from tau with an intact C-terminus, compared to that with an intact N-terminus, indicating that most tau released from AD synaptosomes is C-terminal truncated. This result is consistent with our previous observation that synaptic tau lacks a C-terminus; in synaptosomes, those peptides with a C-terminal end are also generally full length (~55 kDa^50^).

### Tau released from cortical synapses is exosomal

**Fig. 3:**
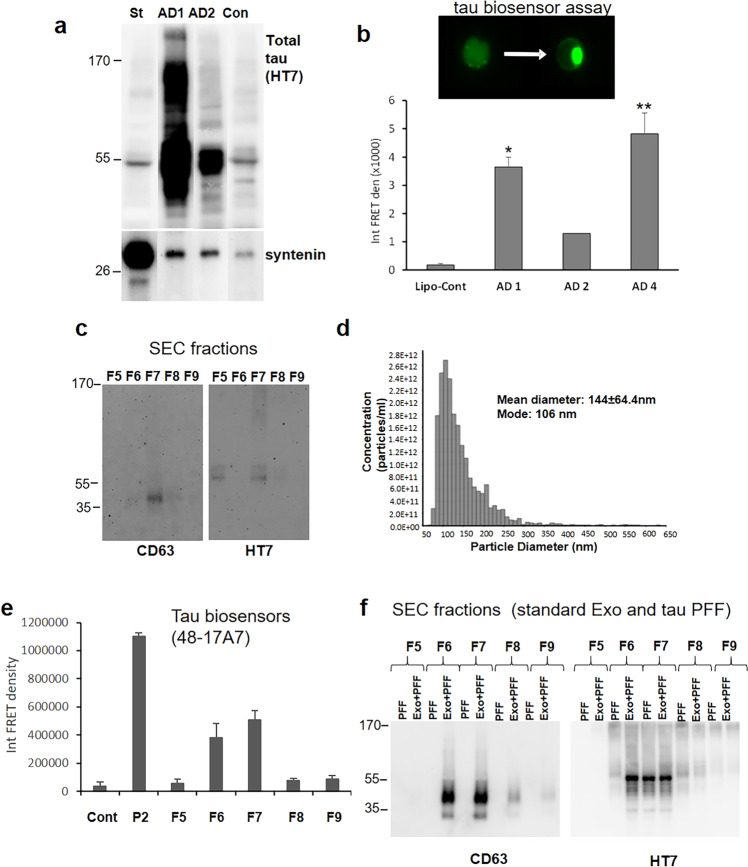
AD cortical synapses release exosomal tau with seeding activity. **a** Large (~200 mg) P-2 samples from two AD and one control (Con) cases were depolarized and exosomes from each case were purified by simultaneous immunoprecipitation (IP) with antibodies to CD63, CD9, and CD81 (pan exosome isolation). IP samples were immunolabeled with the HT7 antibody against tau and the exosome marker syntenin; the left lane control (St) shows labeling with commercial standard human plasma exosomes. **b** To determine seeding activity, release supernatants from three P-2 samples were concentrated and loaded to HEK293T Tau RD P301S FRET biosensor cells (tau biosensor). Aggregate data (mean ± SEM) are shown for the three AD cases along with lipofectamine control (lipo-cont), all in duplicate; *p < 0.05, **p < 0.01, Students *t* test for independen*t* samples. **c** Representative Western SDS PAGE analysis of SEC fractions shows EV/exosome signal with antibody to tetraspanin CD63 and the total tau antibody HT7. **d** Representative tunable resistive pulse sensing (TRPS) analysis shows the size of particles in F7 fraction consistent with exosomes. **e** HEK293 tau biosensor assay for SEC fractions; integrated FRET density, Int FRET den, Cont is lipofectamine control, P-2 is crude synaptosome positive control. Error bars represent mean ± SEM, *p* < 0.05. **f** Western SDS PAGE of SEC fractions with added standard exosomes (Exo) plus commercial tau fibrils (PFF) alternating with added PFF alone.

Because tau released from synaptosomes can be free-floating and/or exosomal, we next verified exosomal localization of tau. Starting with larger P-2 samples (~200 mg compared to 26 mg) from three AD cases, samples were depolarized as described above and supernatants were collected. Exosomes were purified from each release supernatant using immunoprecipitation with antibodies to the exosome-associated tetraspanins CD63, CD9, and CD81. Beads were separated by magnet and IP samples were immunoblotted with the HT7 antibody against tau. Commercial standard exosomes from healthy adult plasma were included as a positive control. The Western blot showed a strong band for full-length tau (~55 kDa) in two of the three cases (Fig. 3a), with a dark smear of fragments and oligomers in one case (AD1). As noted in Table 1, Case AD1 (3-13) showed advanced AD with diffuse Lewy bodies. Case AD2 (5-13), with strong ~55 kDa bands, was also an advanced AD case, with vascular dementia characteristics noted. The control case (Con; 830), with very little exosomal tau (Fig. 3a), showed little tau pathology (Braak II), no beta-amyloid pathology in parietal cortex, and severe atherosclerosis. This case was included as a control due to the relatively minimal neuropathologic change and the need for a large tissue volume. It is interesting that the standard plasma exosomes (lane 1, St), also have a small tau band at ~55 kDa. The blot was reprobed for syntenin-1, a marker of syndecan–syntenin–alix-dependent pathway of exosome biogenesis^51^, and suggested as a specific marker for small EVs representing “bona fide exosomes”^9^. The density of the syntenin-1 band from synaptic release is very small compared to that from healthy plasma control standard exosomes (lane St, left); together, these results clearly verify exosomal localization of tau peptides released from human AD synapses.

### Synaptically released tau demonstrates seeding activity

In order to test seeding potential of synaptic tau, we depolarized a series of three P-2 samples, starting again with relatively large P-2 sample sizes. Experiments used the FRET tau biosensor assay developed by Diamond and colleagues, which can detect seeding activity in samples before histopathological stains using flow cytometry analysis of HEK293 cells^52^. To eliminate smaller tau peptides that are free-floating, we concentrated each sample to about 20 μl using Vivaspin centrifugal concentrators with a molecular weight cutoff of 100 kDa. Figure 3b shows the seeding potential of 3 AD cases compared to control; note that the AD1 and 2 cases used for Fig. 3b are the same cases as AD1 (Table 1 case 3-13) and AD2 (Table 1 case 5-13) from Fig. 3a. Case AD1 showed much higher exosomal tau and tau oligomer levels compared to AD2, and demonstrated significantly higher seeding activity. There was not sufficient sample to include case AD4, which had the highest seeding activity, in the previous exosome immunoprecipitation experiment (Fig. 3a).

**Fig. 4:**
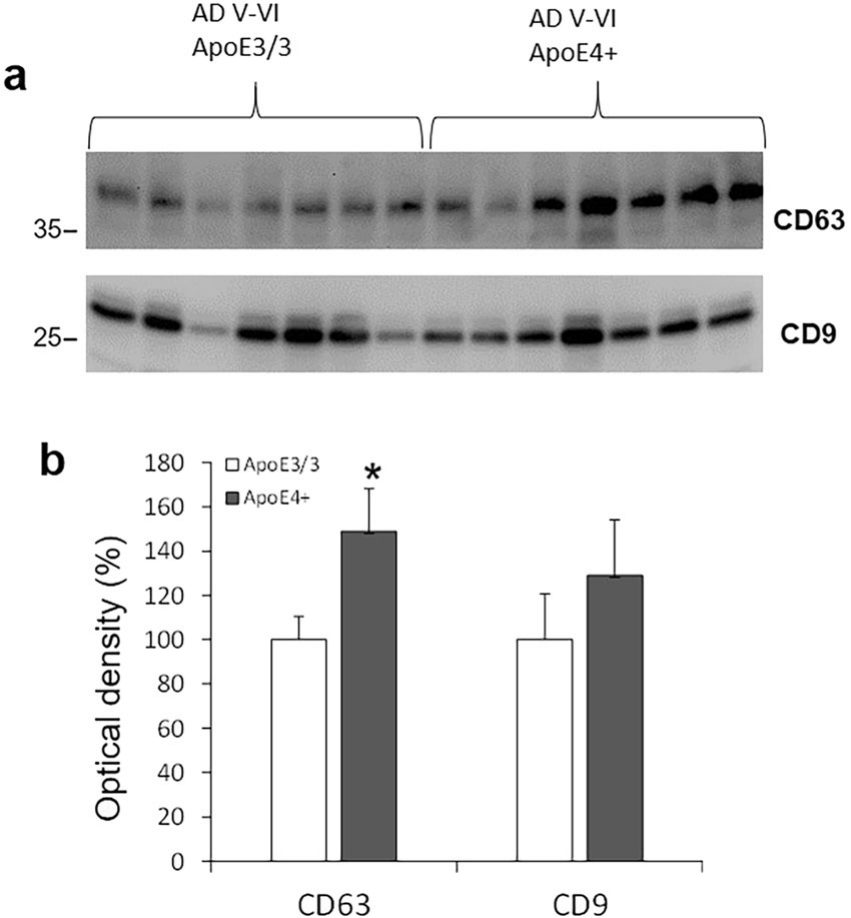
Synaptically released exosomes are increased in cortex of APOE4 compared to APOE3. **a** Exosomes from release supernatants were purified and separated by SEC as described in “Materials and methods”. Western SDS PAGE analysis of the tetraspanins CD63 and CD9; **b** quantification for both tetraspanins; *n = 7, *p* < 0.05.

In order to compare the seeding potential of exosomal and free-floating tau, we initially attempted to concentrate the flow-through from the pan exosome IP experiment above (Fig. 3a), but no seeding potential was detected despite multiple efforts with large samples. Therefore, we next purified exosomes from the release supernatants and separated fractions (F) by SEC. Synaptosomes of AD cases were depolarized as described above, and the release supernatants collected. Exosomes were purified by ultracentrifugation and separated on SEC columns. Fractions 5–9 were collected and Western analysis showed the tetraspanin CD63 and tau in F6 and F7; the tau bands were very faint bands for tau monomer and a HMW tau oligomer at the top of the gel (Fig. 3c). A faint tau monomer band was sometimes observed in fractions other than F6 and F7; for example in fraction F5 (Fig. 3c). These bands most likely represent free-floating tau, since seeding activity was never observed in any fraction besides F6 and F7. In some experiments, CD63 and seeding activity but not tau bands were visible, consistent with loss of the very weak tau signal by dilution and separation with SEC. The size analysis (Fig. 3d) verifies a mean particle diameter with a mean of 144 and a mode of 106 nm, consistent with exosomes. Tau seeding activity in HEK293 cells was also concentrated in fractions F6 and F7 (Fig. 3e). The localization of tau and exosomes to SEC fractions F6 and F7 was confirmed by a control experiment using commercial tau fibrils and standard human plasma exosomes; commercial tau fibrils (PFF) were run in alternating lanes with standard exosomes plus PFF (Fig. 3f). A comparison of released exosomes from *APOE3* and *APOE4* cases is shown in Fig. 4a; seeding activity did not differ by genotype (not shown), although Western analysis of CD63 from F6 indicates increased exosome release in *APOE4* cases (Fig. 4b). The tetraspanin CD9 showed a similar trend that was not significant. Taken all together, these experiments demonstrate the colocalization of seeding activity, exosomes, and tau to fractions F6 and F7, demonstrating that the seeding activity of synaptically released tau is associated with exosomal but not free-floating tau.

### Tau seeding potential is reduced in pure tauopathy cortex compared to AD, but enhanced by amyloid beta

**Fig. 5:**
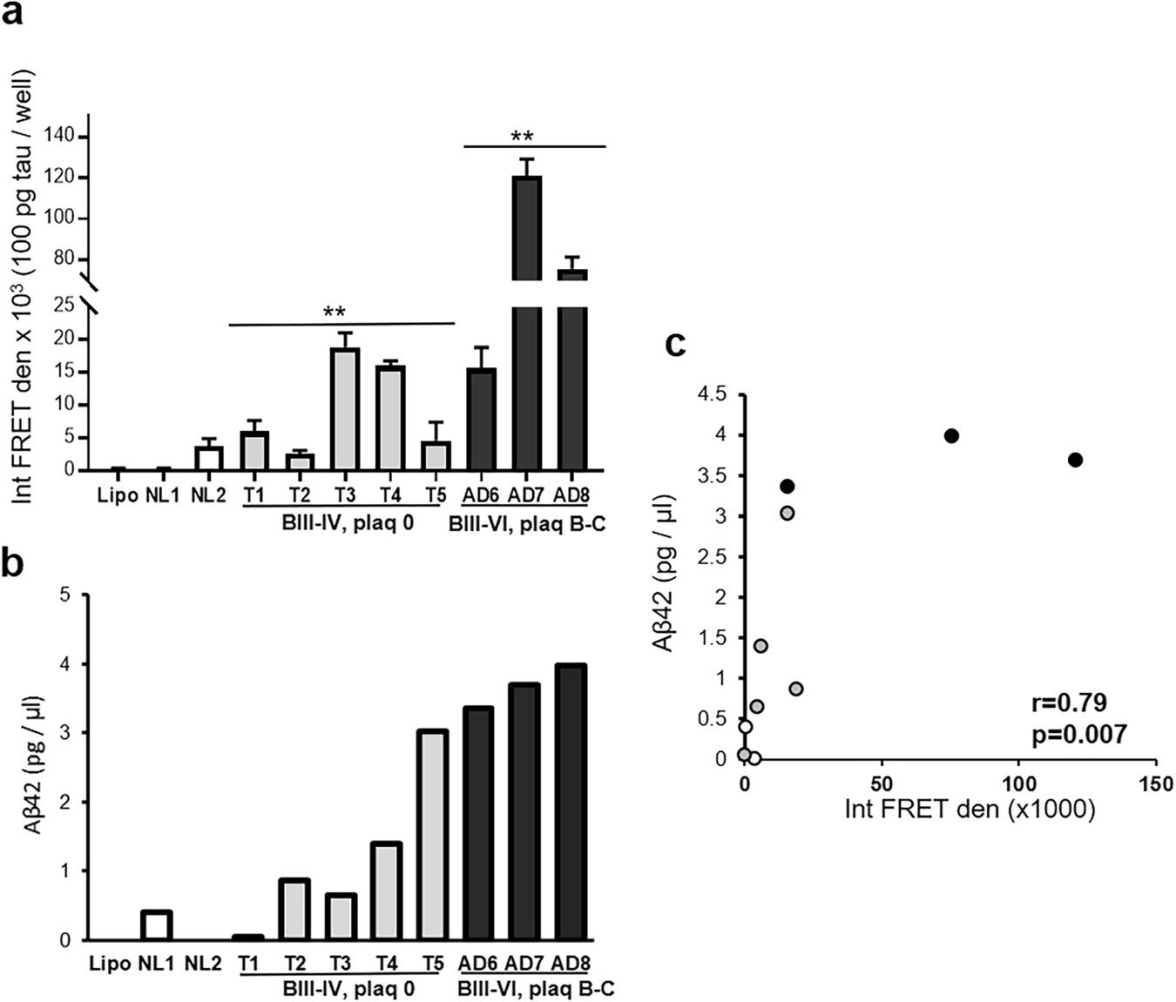
Tauopathy samples demonstrate low seeding potential compared to AD. **a** Tau biosensor cells were seeded with aliquots of cortical P-2 from normal controls (NL1, NL2; white), tauopathy cases without plaques (Braak III–IV, plaque 0; T1–5; gray), and AD cases (Braak III–VI, plaque B,C; AD6-8; black), with lipofectamine control (Lipo). Integrated FRET density (Int FRET den). Seeding activity was increased in tauopathy and in AD compared to controls; **p < 0.01, one-way ANOVA. **b** Soluble Aβ42 was measured in the P-2 from the same cases used in (**a**). **c** Correlation analysis of Aβ42 level and integrated FRET density.

We next asked how seeding activity of tauopathy cases without amyloid plaques compares to seeding activity in AD cases with plaques and tangles, when tau concentrations are similar. For these experiments, cortical synaptosomes (P-2) were used as seeds, since, as noted above (Fig. 3b, e), the seeding activity of synaptosome release fractions is small and requires substantial amounts of starting material. Two aged cognitively normal parietal cortex samples were used as controls; to avoid confounds related to amyloid, five tauopathy cases were selected with no plaque pathology in parietal cortex, and Braak III–IV tau pathology (i.e., no neortical tau). Three AD cases with both tau and plaques in the parietal cortex were included. Soluble P-2 extracts were prepared, and both tau and Aβ42 were measured by alphaLISA immunoassay; extracts were then applied to HEK293 biosensor cells, adjusting the volume so that each well contained ~100 pg tau for each sample. As expected, the control cases showed little seeding activity; Fig. 5a shows that the tau seeding activity was approximately fourfold higher in the two late AD cases, with plaques and tangles in the cortex compared to the five tauopathy cases. Seeding activity was increased in both tauopathy and AD cases compared to control (Fig. 5a; ANOVA *F*(2,17 = 6.35), *p* = 0.0004). The Aβ42 levels for each case are shown in Fig. 5b; even in the absence of plaques, there were variable low-to-moderate levels of Aβ42 in one of the normal (NL1, Table 1 case 1-13,) and the tauopathy cases, consistent with a physiological role for Aβ peptides in regulation of synaptic function^53^. Case AD6 (Table 1 case 7-11) showed fourfold less seeding activity than the other AD cases; this AD case had cognitive impairment but no dementia and intermediate pathology: Braak III, with no tangles but moderate Aβ42 plaques in parietal cortex. The Aβ42 level of the three AD cases did not differ significantly, and all three were rated neuritic plaque level B-C by the neuropathologist; however, the seeding capacity of the two late stage cases, AD7 (Table 1 case 23-11) and AD8 (Table 1 case 12-12), jumped fourfold (Fig. 5c). Even with the very low measured levels of Aβ42 in the tauopathy cases with no plaques, seeding activity showed a significant correlation with the Aβ42 level (*r* = 0.79, *p* = 0.007; Fig. 5c); but not with total tau (*r* = 0.08; not shown). The correlation with Braak & Braak stage was also not significant (*r* = 0.56; not shown). Taken together, these results indicate that tau seeding activity is strongly associated with Aβ42.

## Discussion

A large literature provides direct support for the trans-synaptic spread of tau pathology in AD, but virtually all studies use in vitro or animal models. In the present work, using human cryopreserved cortical tissue throughout, we developed a flow cytometry assay to quantify depolarization of human synaptosomes. Reduced fluorescence from the membrane dye FM2–10 verifies KCl-induced depolarization, and the viability dye calcein AM shows that depolarized synaptosomes remain intact. TEM images show exosome-sized particles in release supernatants, and markers of multiple EV-associated tetraspanin proteins are increased by depolarization. Antibodies indicate that some released tau is phosphorylated and oligomeric, and that most released tau is C-terminal-truncated. Immunoprecipitation with tetraspanin proteins (CD9, CD63, and CD81) definitively localized significant tau peptides and oligomers onto EVs. Importantly, when synaptic release supernatants were fractionated by size, the exosome marker CD63 and tau were observed in the only fractions to demonstrate seeding activity. This indicates that exosomal rather than free-floating tau is the basis for transynaptic spread of tau pathology. In tau seeding experiments with crude synaptosomes (P-2) from control, tauopathy, and AD cases, seeding activity was highly associated with the low levels of Aβ42 detected in tauopathy cortex samples without plaques. However, in these cases, seeding was fourfold less than the maximum seeding activity measured when both plaques and tangles were present in AD cortical samples, indicating that Aβ makes a major contribution to seeding activity.

It is important to note that the FM2–10 assay shows robust depolarization, and that the degree of depolarization in fresh mouse synaptosomes was identical to the cryopreserved human synaptosomes. It is also clear that tau and exosome release occurs at rest in control buffer and the increase with KCl depolarization is fairly modest but significant. We attempted a more thorough characterization of released tau peptides, but it is important to emphasize the extremely low protein and tau levels in the in vitro release supernatants. For example, acetylated tau, caspase-cleaved tau, and additional p-tau epitopes (PHF1, PS396) were not detected (not shown). In the present results, release of fragments and various tau oligomers was observed, especially at about the size of trimers, and exosomal tau from one AD cortical sample (Fig. 3a) showed a dense laddering and smearing pattern above and below the molecular weight of the intact protein at ~55 kDa. This is similar to previous observations by us and others^21,22,54^, and is consistent with accumulation and extensive aggregation of multiple tau fragments rather than one or several key tau cleavage fragments.

Our finding that truncated tau is released would be expected based on our previous result that most synaptosomal tau is C-terminal-truncated^50^; this result is consistent with some observations but there is no consensus in the literature. For example, free-floating-truncated tau was released from human induced pluripotent stem cell (iPSC)-derived neurons, while full-length tau was detected in exosomes^13^; Wang and colleagues also found exosomal tau to be full length^6^. Most human tau secreted by Hela cells was C-terminal-truncated^55^, and several in vitro studies suggest that C-terminal-truncated tau and full-length tau are secreted by different mechanisms^56,57^, although another group, also using cultured neurons, found intracellular tau to be full length, while the majority of extracellular tau was truncated^17^.

Several papers note that only a small fraction of tau is exosomal, with the vast majority of extracellular tau free-floating^6,13^, and free-floating tau assemblies equal to or greater than trimers have been shown to seed aggregation^18^. In the present work, we were unable to seed with concentrated free-floating tau extracts despite multiple efforts. However, SEC separation of released exosomes clearly shows that CD63 and tau are associated with the two fractions that demonstrate seeding activity. The small monomeric tau signals seen occasionally in other fractions, probably representing free-floating tau, were not associated with seeding activity. Along this line, in some experiments, only very faint HMW tau oligomers were present in Fractions 6 and 7, and in some experiments, the tau signal was not visible in those fractions but CD63 and seeding activity were observed. Since others have shown seeding from extracellular tau, it may be that there is less free-floating tau and more exosomal tau from human cortical synapses. It is also possible that very low levels of HMW free-floating aggregates with high seeding potential^19,20^ were present below the assay threshold for seeding activity, an important point since the argument has been made that the HEK biosensor assay represents a non-physiologic form of aggregation^58^. More work is needed to address this issue, but our inability to show seeding activity of the flow-through containing free-floating tau, and the colocalization of released tau and exosomes in fractions with seeding activity is most consistent with the hypothesis that tau spread is exosomal in human AD.

With respect to phosphorylated tau, multiple p-tau epitopes and high levels of p-tau have long been observed in AD tissue and in multiple mouse models, and are detected in synaptosomes^21,59,60^, but there is general agreement in the literature that extracellular tau is relatively hypophosphorylated in multiple model systems. In the present experiments, the released p-tau signal was very faint and only a single p-tau epitope was detected. Similar to our results, in rTg4510 tau transgenic mice, Polanco and colleagues found exosomal tau to be weakly phosphorylated compared to the multiple relatively abundant disease-associated epitopes found in their mouse model^12^. Clear hypophosphorylation of exosomal compared to cytosolic tau was also observed by Wang et al. in N2A cells and primary culture^6^. A potential mechanism is suggested by a similar pattern observed in 3xTg-AD slice cultures and rat cortical neurons, where dephosphorylated tau was observed in membrane-associated fractions^61,62^. These authors suggest that a pool of releasable tau peptides at the membrane awaiting secretion is regulated by phosphorylation. Tau also can be dephosphorylated after release by tissue-nonspecific alkaline phosphatase^63^, but dephosphorylation seems unlikely to affect tau inside EVs. However, a thorough study with human postmortem samples showed that HEK biosensor seeding activity was highly variable between cases, and was associated with disease progression and with phosphorylation on Thr231 and Ser235 or Ser262 but not with other p-tau epitopes^64^. As noted above, we did not detect multiple p-tau epitopes, but given the barely detectable tau in our release supernatants, it is entirely possible that relevant phosphorylated peptides were below the detection limit of our assays.

Our results suggest a higher level of exosome release in the presence of *APOE4*, which might be expected, since exosomes are suggested as a mechanism for export of pathologic proteins. This is in contrast to a previous observation by Peng and colleagues, who demonstrated reduced exosome content in human samples and a mouse model expressing human apoE. The samples used were also cortical samples, but no AD was present, and EVs were isolated using enzymatic dissociation and ultracentrifugation^65^. These authors hypothesized reduced exosome biogenesis in the setting of *APOE4*, possibly contributing to AD risk. In contrast, the present study focused on AD samples and considered only synaptically released exosomes, likely to be responsible for the different results. It is also entirely possible for exosomes to have dual or multifocal actions in AD^66^.

Though the literature for exosomal tau is far more robust, a number of papers clearly show Aβ as an exosome cargo responsible for pathological transfer of Aβ via exosomes^24,59,67,68^, so we were surprised that the extracellular Aβ signal in our AD samples was extremely faint. We did not examine our IP-isolated exosomes from synaptosome release for Aβ (Fig. 3a), because there was a vanishingly small yield, and exosomal Aβ would not be expected, given the low level of total extracellular Aβ in Fig. 2. In one study, with a design similar to ours, Bennett and colleagues found that homogenates from AD brain regions with and without amyloid plaques showed increased seeding potential by two- to threefold, using the tau biosensor assay developed by Diamond and also used by us^69^. Similar to our result in which Aβ but not tau level correlated with seeding activity, these authors concluded that bioactivity rather than level of tau was increased by Aβ, and that soluble Aβ, not just plaque Aβ, was capable for enhancement^69^.

Along this line, in previous work we have shown that the level of synaptic p-tau is markedly higher in individual terminals positive for Aβ; using samples from hippocampus and entorhinal cortex in early stage disease samples (≤Braak IV), we demonstrated a highly significant correlation between early terminal levels of p-tau and Aβ^60^. We concluded that Aβ may directly drive tau phosophorylation early in the disease process, consistent with synaptic p-tau induction that is directly driven within the terminal by protein–protein contact between synaptic oligomeric Aβ and tau^60^. Such a prion-like mechanism occuring within synapses finds strong support in a paper by Vasconcelos et al., which showed heterotypic seeding of tau fibrillization by aggregated Aβ in a cell-free system^70^. As noted by these authors and by others^22,69–72^, prion-like seeding of tau by Aβ explains a number of peculiarities of AD compared to other tauopathies, including the initial appearance of Aβ in isocortical regions^73^, and the clear Aβ dependence of tau-associated cognitive loss^74–76^. Our tau seeding experiments from tauopathy and AD cases (Fig. 5) used P-2 samples; we have previously shown that the vast majority of synaptic Aβ42 is oligomeric^77^. Interestingly, the Aβ42 level in the three AD cases is not significantly different, but the seeding activity for the two late stage cases AD2 and AD3 (Braak V, VI) jumps approximately fourfold from the level of activity of the Braak III stage AD1, a case with light pathology but without dementia (MMSE 26). This result highlights the strong correlation of tau pathology with cognition. However, the mechanism for the seeding activity jump is not clear although it illustrates the clear tau pathology acceleration by Aβ seen by others^22,69,70,78^. It is possible that the Aβ42 concentration has a sharp threshold for seeding activity; perhaps more likely, the sharp uptake is related to another Aβ peptide that correlates with Aβ42, perhaps oligomers or acetylated tau^79^.

The overall correlation between synaptic Aβ42 level and biosensor activity is strong. Taken together with our earlier data showing more p-tau in Aβ-positive synapses^60^, and previous work by others^70,80^, the direct templating of misfolded tau by Aβ oligomers within synapses may represent a plausible mechanism for the Aβ boost in tau propagation. We cannot exclude that seeding activity is also driven by differences in tau conformers^81,82^, which is supported by a number of papers showing prion-like spread of misfolding between tau peptides^83–87^. On the other hand, a number of studies have clearly demonstrated that the presence of Aβ accelerates tau aggregation, propagation, and cellular toxicity^22,69–71,78,88–90^ supporting a hypothesis that differences in terminal Aβ amyloid load may be the primary reason for observed differences in tau seeding activity.

The extremely low levels of extracellular Aβ in our results argues against the possibility that aggregated Aβ directly templates misfolded tau within exosomes as a major mechanism, although Aβ/tau direct contact might occur. Our data are most consistent with a model where misfolded aggregated tau, accelerated by terminal amyloid levels, would subsequently be loaded to exosomes and exported from the synapse, resulting in exosomal transfer of tau across synapses. More work is needed, but such a model is consistent with our data showing aggregated exosomal tau, the failure of free-floating tau to seed, and multiple reports by us and others of synaptic accumulations of misfolded, fibrillary tau^22,24,69,91,92^. The hypothesized model would support the consensus that amyloid is the upstream initiating pathology, and the observed failure of anti-amyloid therapies even when given early. This model, where Aβ accelerates the aggregation of synaptic tau, may provide support for aggregation inhibitors^93,94^ and tau-targeted therapeutic approaches, including a reduction of secretion of tau-bearing exosomal subpopulations^26,95^.

## Data Availability

All the data generated or analyzed during this study are included in the manuscript.
